# Recent Advances in the Electrochemical Sensing of Venlafaxine: An Antidepressant Drug and Environmental Contaminant

**DOI:** 10.3390/s20133675

**Published:** 2020-06-30

**Authors:** Somayeh Tajik, Hadi Beitollahi, Zahra Dourandish, Kaiqiang Zhang, Quyet Van Le, Thang Phan Nguyen, Soo Young Kim, Mohammadreza Shokouhimehr

**Affiliations:** 1 Research Center for Tropical and Infectious Diseases, Kerman University of Medical Sciences, Kerman 76169-11319, Iran; s.tajik@kmu.ac.ir; 2 Environment Department, Institute of Science and High Technology and Environmental Sciences, Graduate University of Advanced Technology, Kerman 76311-33131, Iran; h.beitollahi@kgut.ac.ir (H.B.); Z.dourandish@kgut.ac.ir (Z.D.); 3 Jiangsu Key Laboratory of Advanced Organic Materials, Key Laboratory of Mesoscopic Chemistry of MOE, School of Chemistry and Chemical Engineering, Nanjing University, Nanjing 210023, China; zhangkaiqiang126@snu.ac.kr; 4 Institute of Research and Development, Duy Tan University, Da Nang 550000, Vietnam; 5 Laboratory of Advanced Materials Chemistry, Advanced Institute of Materials Science, Ton Duc Thang University, Ho Chi Minh City 700000, Vietnam; nguyenphanthang@tdtu.edu.vn; 6 Faculty of Applied Sciences, Ton Duc Thang University, Ho Chi Minh City 700000, Vietnam; 7 Department of Materials Science and Engineering, Korea University, 145 Anam-ro Seongbuk-gu, Seoul 02841, Korea; 8 Department of Materials Science and Engineering, Research Institute of Advanced Materials, Seoul National University, Seoul 08826, Korea; mrsh2@snu.ac.kr

**Keywords:** venlafaxine, modified electrode, voltammetry, electrochemical sensor

## Abstract

Venlafaxine (VEN), as one of the popular anti-depressants, is widely utilized for the treatment of major depressive disorder, panic disorder, as well as anxiety. This drug influences the chemicals in the brain, which may result in imbalance in depressed individuals. However, venlafaxine and its metabolites are contaminants in water. They have exerted an adverse influence on living organisms through their migration and transformation in various forms of adsorption, photolysis, hydrolysis, and biodegradation followed by the formation of various active compounds in the environment. Hence, it is crucial to determine VEN with low concentrations in high sensitivity, specificity, and reproducibility. Some analytical techniques have been practically designed to quantify VEN. However, electroanalytical procedures have been of interest due to the superior advantages in comparison to conventional techniques, because such methods feature rapidity, simplicity, sensitivity, and affordability. Therefore, this mini-review aims to present the electrochemical determination of VEN with diverse electrodes, such as carbon paste electrodes, glassy carbon electrodes, mercury-based electrodes, screen-printed electrodes, pencil graphite electrodes, and ion-selective electrodes.

## 1. Introduction

Environmental problems caused by the large usage of antidepressants have gained wide attention in scientific communities. Anti-depressants are released into the environment by human metabolism, followed by excretion, the discharging of unwanted or expired active compounds from lavatories or trash, and even from wastewater of hospitals or pharmaceutical factories [1]. Anti-depressants are often needed for long-term treatment, thus they are more largely produced in comparison to other medications.

However, the increasing utilization of the anti-depressants and their emergence in municipal wastewater has led to potent behavioral and physiological impacts on aquatic organisms [2,3].

According to studies, Venlafaxine, 1-[2-(dimethylamino)-1-(4-methoxyphenyl) ethyl] cyclohexanol (VEN) is considered one of the new phenethylamine bicyclic anti-depressants with a neuropharmacological profile distinctive from the available anti-depressants, like tricyclic compounds. Moreover, it features the selective inhibition of serotonin and norepinephrine reuptake, and slightly suppresses dopamine reuptake with no considerable affinities for histaminergic, α_1_-adrenergic, as well as muscarinic receptors [4].

VEN has been considered to be administered as a racemate containing S-(+)- and R-(−) enantiomeric forms in the same content. The two enantiomers exhibit pharmacological activities and interactions with diverse signal molecules in the central nervous system. R-enantiomer has been regarded as one of the potential inhibitors of noradrenaline and serotonin reuptake, whereas S-enantiomer features higher selectivity to suppress the serotonin reuptake. It is metabolized in human livers through the cytochrome P450 (CYP) enzymes to one major active metabolite O-desmethylvenlafaxine (ODV, 56%) and two other minor metabolites N, O-di-desmethylvenlafaxine (DDV, 16%) and N-desmethylvenlafaxine (NDV, 1%) [5,6]. Moreover, a significant contribution of ODV in the treatment-impacts of VEN is confirmed due to its existence in plasma at the increased concentrations, as well as a longer half-life (11 to 13 h). The routine dosage ranges from 75 to 375 mg per day [7]. Overdosing symptoms involve depression of the central nervous system, cardiac arrhythmia, hypotension (low blood pressure (LBP)) or hypertension (high blood pressure (HBP)), coma, death, and serotonin syndrome. As VEN has been considered an antidepressant drug, with the highest rate of prescription throughout the world, stereo-selective detection of VEN concentration and its metabolite is very promising to further the knowledge of the mechanisms of enantiomers and the respective pharmacokinetic and pharmaco-dynamic features [5,6,8,9]. Hence, the reliable and sensitive analytical techniques for the determination of VEN are crucial requirements to examine this analyte in various kinds of the specimens with complicated matrices.

According to studies in this field, the commonly utilized procedures to analyze VEN in the biological samples are based on the high-performance liquid chromatography (HPLC) with ultra-violet detection, liquid chromatography/mass spectrometry (LC/MS), gas chromatography (GC), capillary electrophoresis, solid-phase extraction, and tandem mass spectrometry (LC–MS/MS), followed by spectrofluorometric determination [10]. Nevertheless, these procedures suffer from many caveats. These methods would be expensive and laborious. Moreover, in addition to large solvent use, it is necessary to pretreat the specimens. As a result of the electroactive features of VEN, this drug could be detected via electrochemical procedures. VEN electroanalysis is based on VEN electrooxidation on the surface of the electrode.

In comparison to many analytical procedures, advancements in the electrochemical technique include the increased selectivity and sensitivity in determining analytes of organic molecules and medicines without the need for pretreatment or pre-separation. Moreover, it features benefits like inexpensiveness, shorter analysis duration, wider linear responses, acceptable stability, greater accuracy, and reproducibility. Sensors based on current measurements (voltammetric methods) are the most common in use. Current measurement-based sensors are characterized by applying a potential to a working electrode vs. a reference electrode and measuring the current. The current is a result of electrolysis due to the electrochemical reduction or oxidation at the surface of the working electrode. However, this process depends not only on the mass transport rate of molecules to the electrode, but also on the electron transfer rate at the electrode surface. Furthermore, it was found that voltammetric procedures like cyclic voltammetry (CV), square-wave voltammetry (SWV), and differential pulse voltammetry (DPV) have high sensitivity to determine organic molecules such as the drugs and pertinent molecules in the pharmaceutical dosage forms and biological fluids [11,12].

The chemically modified electrodes (CMEs) were intensively investigated while studying the electrocatalytic reaction of diverse analytes, such as pharmaceutical analytes. The CMEs offered distinct benefits in comparison to the unmodified electrodes. Moreover, they include catalytic oxidation or reduction, reflecting higher over-potentials in the unmodified electrodes, which lowers the peak potential. The CMEs enhanced sensitivity and improved selectivity in pharmaceutical analyses [13].

Electroanalytical chemistry has experienced many advancements in developing modified carbon-based electrodes, screen-printed electrodes, ion-selective electrodes, and mercury-based electrodes. The present review attempted to present a critical review on the existing studies addressing the current electrodes, modified electrodes, the respective methodologies, and their utilization. Additionally, we aimed at providing an overview of the new approaches to the design of electrochemical systems and instruments to determine VEN.

## 2. Electrodes

It is widely accepted that a characteristic electrochemical sensor contains a sensing electrode (known as working electrodes), a reference electrode (commonly saturated calomel electrode or Ag/AgCl electrode), as well as a counter electrode, separated by a thin layer of an electrolyte. Electrochemical measurements are usually employed for the detection of electroactive species, based on the modulations of the electrical properties of the analytes that undergo redox reactions [14].

The redox reaction process takes place in working electrodes; the electrochemical sensors are based on potentiometric, voltammetric or amperometric measurements. For potentiometric sensors, a local equilibrium is established at the sensor interface, where either the electrode or membrane potential is measured, and information about the composition of a sample is obtained from the potential difference of the two electrodes. There are three basic types of potentiometric devices: ion-selective electrodes, coated wire electrodes, and field-effect transistors. Amperometric sensors exploit the use of a potential applied between the reference and working electrode, causing the oxidation or reduction of an electroactive species; the resultant current is measured. On the other hand, a voltammetric measurement is made when the potential difference across an electrochemical cell is scanned from a preset value to another; the cell current is recorded as a function of the applied potential. In both cases, the essential operational feature of voltammetric or amperometric devices is the transfer of electrons to or from the analyte. The basic instrumentation requires potential-controlled equipment and the electrochemical cell consisting of two electrodes immersed in a suitable electrolyte. The performance of amperometric sensors is strongly influenced by the working electrode material [15,16,17,18].

Furthermore, experts in the field presented diverse electrochemical sensors based on various electrodes such as carbon paste electrodes (CPE), glassy carbon electrodes, screen-printed electrodes, mercury-based electrodes, pencil graphite electrodes (PGE), and ion-selective electrodes for determining VEN. Out of numerous kinds of the carbon electrodes, CPE was initially proposed by Ralph Norman Adams nearly 60 years ago. The researchers considered the CPEs a common electrode for sensing applications because of their renewability, inexpensiveness, stable responses, and lower Ohmic resistance in comparison to other solid electrodes, minor background currents, and wider potential windows. Additionally, CPEs have been considered as a simple conductive matrix to procure CMEs. Modified CPEs feature acceptable electrocatalytic activities, selectivity, sensitivity, and lower limit of detection (LOD) than the former CPEs. CPE belongs to the non-toxic and environmentally friendly electrode family and has widespread utilization for voltammetric measurements. Notably, oil or aqueous solution interfaces and three-phase junctions (electrode/oil/aqueous electrolyte) contributed importantly to the effectiveness in the electrochemical features and CPE sensitivity [19,20].

According to studies within the field, glassy carbon (GC) has been considered as a type of non-graphitic carbon produced by pyrolyzing a specific polymeric precursor. The GC microstructure contains discrete pieces of the curved carbon planes such as incomplete fullerene-associated nanoparticles (NPs). Its synthesis at the increased temperature of less than 2000 °C exhibited a network of stacked graphite-like ribbon molecules. Moreover, the entity of the polyhedral graphite crystals has been determined in the commercial glassy carbon. The network of the tangled and randomly carbon planes form a dense carbon structure. GC has been widely applied as one of the electrode materials for electroanalysis because of its lower reactivity, higher hardness, impermeability, and reasonable electrical conductivity. As a result of the chemical inertness, this material has been frequently utilized as one of the substrates for casting powder catalysts for the evaluation of their catalytic functions in the electrochemical reaction. Nevertheless, carbon oxidation could happen at an increased anodic potential, which causes deterioration of the electrode and potentially influences the assessment of the electrochemical performances of catalysts. Nonetheless, the carbon electrochemical oxidation has benefits for electrochemical sensors, because it is usually accepted that the GC electrochemical oxidation activates the electrode surface, causing more rapid electron transfer kinetics [21].

It is widely accepted that screen-printed electrodes (SPEs) are considered as instruments generated by printing various inks on different kinds of ceramic or plastic substrates [22]. Compositions of diverse inks utilized to print on the electrodes determines the sensitivity and selectivity, which is necessary for all analyses. It has been demonstrated that screen printing would an encouraging technology to fabricate electrochemical sensors, because it enables the large-scale construction of economical, disposable, and reproducible electrodes with an expanded scope for modification. Additionally, modification has been allowed for the design of a single strip three-electrode system, electrode array, ring electrode, and ultramicroelectrode. Lastly, easy surface grafting with the two chemical and electrochemical procedures with the catalytically selective chemicals would lead to more flexible utilizations [23,24].

However, one of the very reproducible stationary spherical mercury electrodes has been produced by developing a hanging drop electrode, reported by Gerischer, Berzins, and Delahay, showing an encouraging option as an analytical tool. They presented a two-electrode system containing a hanging mercury drop electrode and a mercury film electrode, conventionally employed to attain increased sensitivity and reproducibility of the stripping method. Notably, the mercury electrodes centrally contribute to the advances in electroanalytical chemistry due to the mercury surface reproducibility, utilized for anodic stripping voltammetry and direct voltammetry [25,26].

ISEs have become one of the common tools in chemical analyses due to their uses in clinical diagnostics, environmental monitoring, and process control. Considering their mass production as well as measurements in small volumes, researchers considerably investigated ISE miniaturization. In comparison with the remaining electrochemical determination procedures, such as cyclic voltammetry (CV) and impedance, the ISE-based potentiometric determination mechanism would be one of the most simplified instruments because any external excitement such as current and voltage would not be needed during measurements. Moreover, potentiometric readout features the capacity of being the icon of the ion chemical information for the wearable ISE sensors. Regarding these benefits, the ion-selective electrodes would be utilized in analytical chemistry and biochemical or biophysical studies [27,28].

Materials used in PGE would be economical, simply stored and maintained, and plentiful of windows of a wider potential. Expressed differently, the PGEs exhibit unsuitable electrocatalytic sensitivity over many distinct electroactive molecules. Consequently, modifying the PGEs with a proper electrocatalyst would be vital to build sensitive electrochemical sensors. Therefore, for improvements in PGE electrocatalytic features, it is possible to readily modify the surface, with higher electrochemical reactivity and surface areas [29].

## 3. Electrochemical Detection of Venlafaxine

### 3.1. CPEs

Notably, Madrakian et al. presented one of the current molecularly imprinted poly-mercoated magnetite NPs (VENMNPs) to determine VEN. They employed the synthesized NPs as a nano modifier for CPE [10]. Then, this modified electrode was applied as the electrochemical sensor to electroxidize VEN and to determine VEN sensitively and selectively. Afterward, the electrochemical impedance spectroscopy (EIS), CV, and also DPV were utilized for the characterization of the modified CPE. A variety of experimental variables with potent effects on the electrochemical signals of VEN electrooxidation like pH, stirring duration, VENMNP dose, accumulation potential, and time were optimized. Concerning the findings, at the optimized conditions, it would be possible to achieve the VEN sensitive determination in the concentration ranging from 0.01 to 10.0 μM. Moreover, LOD for determining this medicine was 6.0 nM. Ultimately, the outputs of analyses on VEN concentration in the humans urine and blood serum samples revealed the development of a robust candidate procedure to determine the VEN in biological fluids [10].

Another study conducted by Eslami et al. combined the multi-walled carbon nanotubes (MWCNTs) and the ionic liquid, 1-octylpyridinium hexaflouro-phosphate (OPFP), and yielded a carbon nanocomposite (MWCNT-CILE) with an acceptable utilization as the electrode for the VEN electrocatalytic oxidation [30]. These researchers examined the influences of diverse experimental variables like the solution pH, the amounts of MWCNTs utilized to modify the carbon ionic liquid electrode (CILE), accumulation potential and time, and the rate of scan on VEN voltammetric responses. The analyses showed the VEN oxidation peak potential at 640 mV at the new electrode equal to nearly 150 mV lower than the peak potential in the previous CPEs. The oxidation was irreversible and exhibited absorption-controlled behaviors. Based on the optimized condition, the anodic peak current was linearly associated to the VEN concentrations in a range of 10.0 to 500.0 μM. Consequently, LOD (three times signal to noise ratio) equaled 0.47 μM. Ultimately, this new sensor had sensitivity, reproducibility, simple renewability, as well as affordability, suggesting encouraging utilization for determining VEN in drug formulations, blood serum, and urine samples [30].

In another study, Rizk et al. applied the CPE modified with zinc oxide NPs immobilized on the MWCNTs (MWCNTs/ZnONPs/CPE) to simultaneously determine clonazepam (CLZ) and desvenlafaxine succinate (DVS) in the drug materials, the combined drug products, and in spiked human urine [31]. Moreover, DPV and CV were used to investigate electrochemical response features of the modified electrode toward DVS and CLZ. Therefore, CVs were accompanied by the organized irreversible cathodic peaks (reduction) at the potentials of nearly −407 to −636 mV in Britton–Robinson (BR) buffer (pH = 9.0) for DVS and CLZ, respectively. The researchers also examined and optimized diverse experimental variables influencing the procedure sensitivity such as the supporting electrolyte pH, electrode modifier, various surfactants, and the scan rates. Consequently, a satisfactory linear association was observed between the peak current and concentration in ranges of 0.66 to 8.42 μg/mL and 0.39 to 7.70 μg/mL for DVS and CLZ with DPV. Finally, this procedure was substantially utilized to estimate CLZ and DVS in the spiked human urine [31]. Many characteristics of the CPE in VEN electrochemical detection are summarized in Table 1.

### 3.2. GCE

Sanghavi et al. presented a Nafion-carbon nanotube modified GCE (NAF-CNT-GCE) to determine desvenlafaxine (DVF) and VEN [32]. They utilized chronocoulometry (CC), CV, absorptive stripping differential pulse voltammetry (AdSDPV), and EIS for the investigation of the electrochemical behaviors of the molecules. Moreover, the researchers used scanning electron microscopy (SEM) to study the morphology of the electrode surface. Based on the outputs, DVF and VEN oxidation was facilitated at the NAF-CNT-GCE. Upon optimizing the analytical condition, which employed this electrode at pH equal to 7.0 in the BR buffer (0.05 M) for VEN and pH equal to 5.0 in acetate buffer (0.1 M) for DVF, the peak current for the two molecules showed a linear variation with their concentration in the ranges of 5.33 × 10^−8^ to 3.58 × 10^−5^ M for DVF and 3.81 × 10^−8^ to 6.22 × 10^−5^ M for VEN. Furthermore, LODs (S/N = 3) equal to 1.24 × 10^−8^ and 2.11 × 10^−8^ M, respectively, were reported for VEN and DVF with AdSDPV. Notably, this modified electrode featured multiple benefits, like the simplistic procurement procedure, higher sensitivity, low LODs, and good reproducibility. Finally, their new technique was utilized to determine DVF and VEN in the drug formulations, urine, and the blood serum samples [32].

Furthermore, Ding et al. addressed VEN electrocatalytic oxidation over a GCE modified by a gel consisting of the MWCNTs in an ionic liquid (RTIL), 1-butyl-3-methyl-imidazolium hexa-fluorophore (BMIMPF_6_), in 0.10 M PBS (pH equal 6.8) at room temperature [33]. The researchers observed the irreversible anodic oxidation peak of VEN with a peak potential of 0.780 V at the MWCNTs-RTIL/GCE. Hence, the electrode reaction procedure is a diffusion-controlled process and electrochemical oxidation contains two transferring electrons and two participating protons. Consequently, the charge transfer coefficient (α) and constant of the electrode reaction rate (k_f_) of VEN are equal to 0.91 and 3.04 × 10^−2^ s^−1^. Based on the optimum conditions, electrocatalytic oxidation peak currents had a linear dependence on VEN concentration with a concentration range of 2.0 × 10^−6^ to 2.0 × 10^−3^ M with LOD (S/N = 3) equal to 1.69 × 10^−6^ M. Their new method experienced a successful application in the electrochemical quantitative detection of the amount of VEN in commercial VEN hydrochloride capsules, and the detection outputs could satisfy the requirements of quantitative detection [33]. Many characteristics of the GCE in VEN electrochemical detection are summarized in Table 2.

### 3.3. SPE

**Figure 1:**
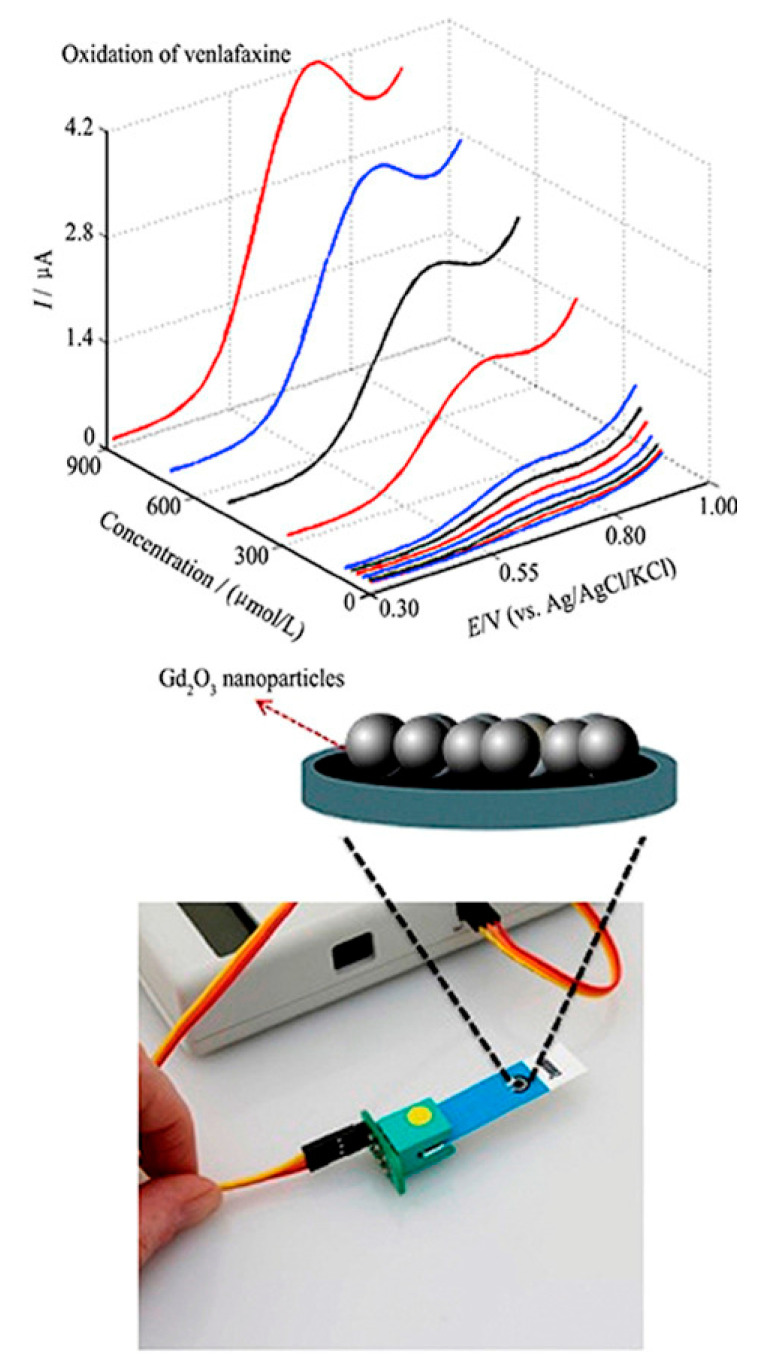
Electrochemical nanosensor for the determination of venlafaxine using Gd_2_O_3_/SPE. Reproduced with permission [3]. Copyright 2019, Elsevier.

Beitollahi et al. designed the graphite screen-printed electrode (GSPE) modified with the Gd_2_O_3_ NPs (Gd_2_O_3_/SPE) to determine VEN as presented in Figure 1 [3]. They showed Gd_2_O_3_ NPs characteristics, which are provided by transmission electron microscopy (TEM), X-ray diffraction (XRD), and SEM. Therefore, for studying CV curves, DPV and chronoamperometry (CHA) electrochemical behaviors were utilized. According to the outputs, VEN oxidation would be easier at the Gd_2_O_3_/SPE. After the analytical condition optimization, analyzing VEN with the modified electrode in 0.1 M PBS (pH = 7.0) demonstrates that the peak currents, which correspond to VEN, varied linearly with its concentration range of 5.0 × 10^−6^ to 9.0 × 10^−4^ M. Moreover, Beitollahi et al. achieved a 0.9994 correlation coefficient and 0.21-μM LOD [3]. Notably, the procured modified electrode featured benefits such as a simplistic preparation procedure, higher sensitivity, and lower LOD. Additionally, the functional utility of the new technique was successfully assessed for the identification of VEN in drug formulations, water, and urine specimens.

The other study by Maddah et al. devised one of the sensitive electrochemical SPEs modified with the La^3+^/Co_3_O_4_ nanocubes for VEN determination, demonstrating higher validity [13]. The researchers synthesized La^3+^/Co_3_O_4_ nanocubes via a simplified hydrothermal procedure based on the optimum synthesis condition and featured through SEM and XRD analyses. They also employed the electrochemical procedures like DPV, CV, and CHA for monitoring VEN. Therefore, rising the oxidation peak current and varying to the less positive potentials than the bare SPE approved the involvement of the modified electrodes in accelerating the reactions. Moreover, the findings obtained from DPV exhibited greater sensitivity in detecting VEN in 1.0–500.0 μM with LOD equal to 0.5 μM (S/N = 3). Finally, this disposable sensor showed a satisfactory utilization in the drug formulations, water, and urine samples [13].

**Figure 2:**
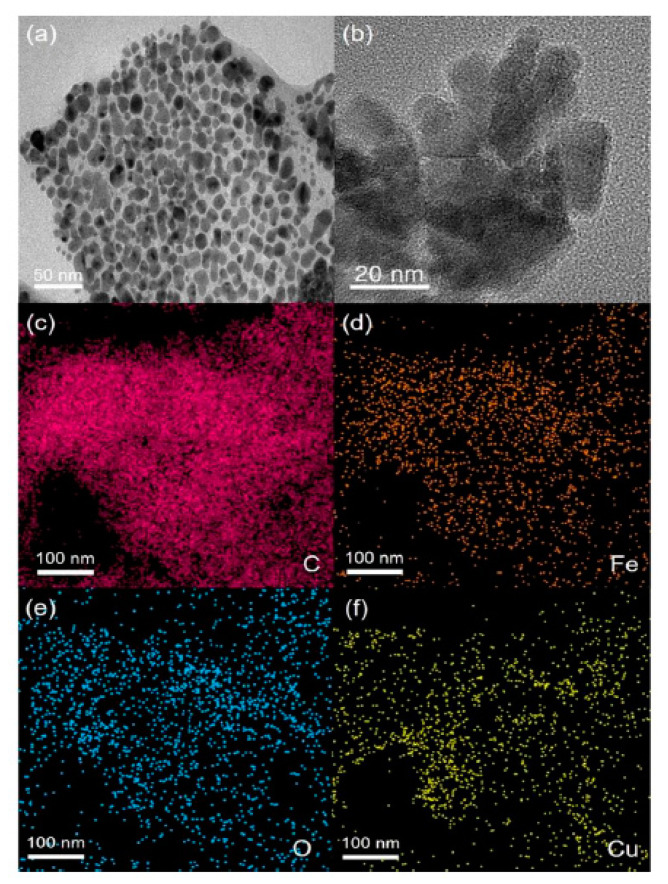
(**a**,**b**) Transmission electron microscopy (TEM), high resolution TEM (HRTEM) images; (**c**–**f**) energy dispersive X-ray (EDX) map of (Fe_3_O_4_@CNC/Cu). Reproduced with permission [34]. Copyright 2020, ACS.

Moreover, Khalilzadeh et al. made a sensitive SPE electrochemical sensor based on the Fe_3_O_4_@cellulose nano-crystals/Cu nanocomposite (Fe_3_O_4_@CNC/Cu) to sensitively determine the VEN [34]. To sum up, a green method with the petasiteshybridus leaf extract as one of the reducing and stabilizing agents of copper was utilized to magnetically synthesize Fe_3_O_4_@cellulose nanocrystals/Cu nanocomposite. The researchers used field emission-scanning electron microscopy (FE-SEM), thermal analysis, UV–VIS spectral analysis, XRD, energy-dispersive X-ray spectroscopy (EDAX), TEM, vibrating sample magnetometer (VSM), and Fourier transforms infrared spectroscopy (FTIR) to validate Fe_3_O_4_@CNC/Cu formation (TEM and EDAX shown in Figure 2). Then, Fe_3_O_4_@CNC/Cu was applied for the modification of a GSPE, which analyzes VEN concentration. The experiments revealed suitable functionality of the electrode for electrochemically determining the VEN. Finally, at the optimum condition, a linear response was obtained for the VEN in a concentration range of 0.05 to 600.0 µM with a LOD equal to 0.01 µM using the modified sensor [34]. A number of characteristics of the SPE in VEN electrochemical detection are summarized in Table 3.

### 3.4. Mercury-Based Electrodes

Lima et al. addressed the VEN electrochemical oxidation over a hanging mercury drop electrode (HMDE) in a buffered aqueous solution with a wider pH-range from 1.9 to 10.0 under diverse potential sweep procedures [35]. Based on the outputs, the most reasonable definition of analytical signals was reported for boric acid: potassium tetra-hydroxoborate buffer at pH equal to 8.7 with the anodic stripping square wave voltammetry (SWASV). The recovery trials were carried out for the assessment of the precision of outputs, which were also compared with the results shown by HPLC based on the procedure reported in the previous investigations and characteristics of the drug formulations. Finally, the relative deviation equal to 0.2% of B and LOD equal to 0.124 mg/L [35].

Additionally, Morais et al. designed one of the absorptive stripping voltammetric procedures to determine the antidepressant VEN in urine with a mercury film microelectrode [36]. Their technique is based on the controlled absorptive accumulation of this medicine at a potential equal to −1.00 V (vs. Ag/AgCl) in the presence of 1.25 × 10^−2^ M borate buffer (pH equal to 8.7). The direct analysis of the urine samples was carried out following a ten-fold dilution with a supporting electrolyte but with no additional pre-treatment. Finally, LOD was equal to 0.693 × 10^−6^ M for a 30-s collection time and testing recovery showed satisfactory outputs at 10^−6^ M (bias >5%) [36]. A number of characteristics of the mercury-based electrode in VEN electrochemical detection are summarized in Table 4.

### 3.5. Various Electrodes (Ion-Selective Electrode, PGE, etc.)

In their study, Ensafi et al. addressed the fabrication and electrochemical response properties of the membrane sensor of polyvinyl chloride (PVC) to determine the VEN [37]. The analyses showed that this sensor contained dibutyl phthalate (DBP) as one of the plasticizers and venlafaxine–sodium tetraphenylborate (ion pair) as one of the electroactive materials in a PVC matrix within a percentage ratio of 5.1: 66.0: 28.9 (ion pair: DBP: PVC, w/w/w). It also linearly responds to the VEN in a concentration range from 1.0 × 10^−2^ to 8.0 × 10^−6^ M with the LOD equal to 5.0 × 10^−6^ M and with a slope equal to 29.4 ± 0.1 mV decade^−1^ in a pH range from 3.0 to 9.0. Researchers addressed the sensor selectivity coefficient for the VEN relative to the number of the potent interfering materials. It was found that this sensor has a great selectivity for the VEN in multiple similar compounds. It also showed a rapid response time of 10 s and was utilized for two months (for two determinations/day) with an acceptable reproducibility. Finally, the researchers observed a successful application for determining the VEN in the urine, blood serum, and pharmaceutical samples with reasonable outputs [37].

Another study conducted by Mansour et al. showed the encouraging future of the ion-selective electrodes to examine the VEN [38], particularly in pharmaceutical preparation and serum with no pretreatment. To sum up, these sensors are based on the utilization of ion association complexes of the VEN cation with silicotungstate (ST) and phosphotungstate (PT) counter anions as the ion exchange sites in a plasticized PVC matrix. The sensors feature characteristics such as conductimetric and potentiometric measurements at different conditions. Moreover, these electrodes exhibited a rapid (response time of ~15 s), linear (r^2^ = 0.995), and stable response (life span of 45 days) for the VEN in a concentration range from 5 × 10^−5^ to 1 × 10^−2^ M venlafaxine hydrochloride. Moreover, the soluble product of the ion pair and formation of the precipitation reaction resulting in the ion pair’s conductimetrical detection showed the high selectivity and accuracy of the electrodes (RSD < 1%) and their applicability for potentiometrically determining the VEN hydrochloride in pure solution or pharmaceutical procurement as well as in biological fluid (serum) with no interferences. Finally, technique validation showed the appropriateness of new electrodes in evaluating the venlafaxine hydrochloride quality [38].

Ali et al. comparatively dealt with the utilization of two distinctive deposition methodologies to synthesize NiCo_2_O_4_@rGO-modified graphite electrode [39]. They used diverse procedures such as EIS, CV, Raman spectroscopy, and SEM to characterize differences between two in situ modified electrodes NiCo_2_-O_4_@rGO-modified electrodes and wet-NiCo_2_O_4_@rGO. The researchers regarded the wet-NiCo_2_O_4_@rGO-modified electrode a simplified and very good sensing tool for utilization in VEN voltammetric detection. Additionally, they examined and optimized various electrochemical variables. Then, the validity of SWV was confirmed based on the ICH instructions, reflecting greater sensitivity (LOD equal to 3.4 nM) with higher accuracy (%RSD < 2.97). Their technique showed a successful application for determining the VEN in pharmaceutical formulations and human plasma. Ali et al. did not observe any interferences from the real sample matrix [39]. Their new electrode exhibited very good reproducibility and excellent stability. Finally, they combined the bi-metallic micro-spheres hybrid and the strongly conductive reduced graphene oxide (wet-NiCo_2_O_4_@-rGO) probe as one of the sensitive and selective tools for VEN trace analysis in various matrices [39]. A number of characteristics of the various electrodes in VEN electrochemical detection are summarized in Table 5.

## 4. Future Outlook

This review features a brief overview of recent advances in electrochemical sensor for VEN detection. Some works, based on various electrodes, are of high quality and have achieved successful applications in VEN sensors because of their interesting designs, comprehensive and detailed study, high sensitivity, and novel electrode materials. Other than achieving higher sensitivity, selectivity, speediness, stability, and reliability, automaticity has always been the driving force for the sensor designs. Nanomaterials are also widely used as carrier beacons for an indirect, yet highly robust and accurate means for detecting target molecules. With the discovery of new nanomaterials, we expect researchers to intensively exploit them for the development of better sensing methods and devices. Nanomaterials provide an enormous signal enhancement due to their large surface–volume ratio, rapid mass transport, facilitated electron transfer, effective catalysis, and strong control over local microenvironment. Moreover, the nanoparticles increase the number of biomolecules immobilized on the surface and make them suitable signal transducers and amplifiers. Electrochemical sensors are both trending products across the sensor market worldwide, and state-of-the-art technologies that exhibit bright prospects in the future. There are still plenty of improvements needed as the quality of the material used in the sensor keeps improving. Engineered materials are the key to enhance the sensing capability in a commonly marketed sensor and to increase the sensitivity of ion selection for a specially purposed sensor.

## 5. Conclusions

Based on the analyses, VEN has been considered as one of the selective serotonin–norepinephrine re-uptake inhibitors utilized for treating depression, generalized anxiety, panic disorder, and social phobia. This drug increases concentrations of the neurotransmitters norepinephrine and serotonin in the brain. Nonetheless, overdosing VEN causes severe consequences and symptoms of serotonin toxicity, depression, cardiac conduction abnormality, and seizure. Therefore, for avoiding such consequences and poisonousness, cautious supervision of the treatment efficacy and the content of medicine in the body fluids is of high importance. Thus, it would be crucial for the experts in the field to devise a simplistic, largely sensitive, and economically-friendly analytical procedure for VEN quantification in real samples. Moreover, the electrochemical techniques provided a robust analytical instrument for sensitively, simply, rapidly, selectively, and affordably determining VEN. Limited studies have been published to examine the usability of electrochemical procedures for determining VEN. Thus, the present review takes action by compiling diverse research strategies to electrochemically detect VEN.

## Figures and Tables

**Table 1:** Many characteristics of the carbon paste electrodes (CPE) in venlafaxine (VEN) electrochemical detection.

Sensor	Method	Limit of Detection	Linear Dynamic Range	Ref
VENMNPs/CPE	DPV	6.0 nM	0.01–10.0 μM	[10]
MWCNT/CILE/CPE	CV	0.47 μM	10.0–500.0 μM	[30]
MWCNTs/ZnONPs/CPE	DPV	0.28 μg/mL	0.66–8.42 μg/mL	[31]

Note: DPV: differential pulse voltammetry; CV: cyclic voltammetry.

**Table 2:** Many characteristics of the GCE in VEN electrochemical detection.

Sensor	Method	Limit of Detection	Linear Dynamic Range	Ref
NAF-CNT-GCE	AdSDPV	1.24 × 10^−8^ M	3.81 × 10^−8^–6.22 × 10^−5^	[32]
MWCNTs-RTIL/GCE	SWV	1.69 × 10^−6^ M	2.0 × 10^−6^–2.0 × 10^−3^ M	[33]

Note: AdSDPV: absorptive stripping differential pulse voltammetry; SWV: square-wave voltammetry.

**Table 3:** A number of characteristics of the screen-printed electrode (SPE) in VEN electrochemical detection.

Sensor	Method	Limit of Detection	Linear Dynamic Range	Ref.
Gd_2_O_3_/SPE	DPV	0.21 μM	5.0 × 10^−6^–9.0 × 10^−4^ M	[3]
La^3+^/Co_3_O_4_/SPE	DPV	0.5 μM	1.0–500.0 μM	[13]
Fe_3_O_4_@CNC/Cu/GSPE	DPV	0.01 μM	0.05–600.0 μM	[34]

**Table 4:** A number of characteristics of the Mercury-based electrode in VEN electrochemical detection.

Sensor	Method	Limit of Detection	Linear Dynamic Range	Ref.
HMDE	SWV	0.124 mg/L	0.25–1.23 mg/L	[35]
MFM	DPV	0.693 × 10^−6^ M	-	[36]

**Table 5:** A number of characteristics of the various electrodes (Ion-Selective Electrode, pencil graphite electrodes (PGE), etc.) in the VEN electrochemical detection.

Sensor	Method	Limit of Detection	Linear Dynamic Range	Ref.
Ion pair:DBP:PVC	Potentiometry	5.0 × 10^−6^ M	1.0 × 10^−2^–8.0 × 10^−6^ M	[37]
Ion-selective electrode	Potentiometry	-	5 × 10^−5^–1 × 10^−2^ M	[38]
NiCo_2_O_4_@rGO Modified PGE electrode	SWV	3.4 nM	5.0–500.0 nmol L^−1^	[39]
